# Supplementary material for: Measuring dimensionality and purity of high-dimensional entangled states
Source: Nat Commun. 2021 Aug 27;12:5159. doi: 10.1038/s41467-021-25447-0 (PMC8397747; doi:10.1038/s41467-021-25447-0)
Supplement: Supplementary file 1 — Supplementary Information [file 41467_2021_25447_MOESM1_ESM.pdf]

Measuring dimensionality and purity of high-dimensional entangled states Supplementary Notes

Isaac Nape *[1], Valeria Rodríguez-Fajardo[1], Feng Zhu[2], Hsiao-Chih Huang[3], Jonathan Leach[2], and Andrew Forbes[1]

[1] *School of Physics, University of the Witwatersrand, Private Bag 3, Wits 2050, South Africa*
[2] *School of Engineering and Physical Sciences, Heriot-Watt University, Edinburgh, EH14 4AS, UK*
[3] *Department of Physics, National Taiwan University, Taipei 106, Taiwan*

Supplementary Note 1 - Dimensionality of Pure States

To quantify dimensionality, we adopt the definition of Schmidt number from Ref. [1] for pure states and later show how it can be used to determine the Schmidt rank [2, 3]. The former, which we shall use in this paper, determines the number of modes needed to describe the state irrespective of the spectrum shape. Consider the Schmidt basis states, $|j\rangle|j\rangle$, spanning a high dimensional Hilbert space for two photons, i.e. $\mathcal{H}\otimes\mathcal{H}$. Using this basis, we can describe a nonseparable entangled state as

$$|\Psi\rangle = \sum_{j=0}^{\infty} \lambda_j |j\rangle|j\rangle, \tag{S1}$$

where $|\lambda_j|^2$ is the probability of detecting the biphoton state $|j\rangle|j\rangle$. The dimensionality of such a state can be obtained from

$$K = \frac{\left(\sum_j |\lambda_j|^2\right)^2}{\sum_j |\lambda_j|^4}. \tag{S2}$$

Examples of various types of distributions for $|\lambda_j|^2$ are shown in Supplementary Fig. 1 for $K = 21$ for the OAM basis where the Schmidt basis modes have the form $|\ell\rangle|-\ell\rangle \in \mathcal{H}\otimes\mathcal{H}$. The distributions are: a square distribution, corresponding to a maximally entangled state within a given ℓ-range,

$$\lambda_\ell = 1/\sqrt{2L+1}, \; |\ell| \leq L; \tag{S3}$$

a Gaussian (normal) distribution

$$|\lambda_{\ell_g}|^2 \propto \exp\left[-\frac{|\ell|^2}{\gamma_G^2}\right], \tag{S4}$$

where γ_G scales with the width of the distribution; a SPDC source [4, 5]

$$|\lambda_{\ell_S}|^2 \propto \left(\frac{2\gamma_S^2}{1+\gamma_S^2}\right)^{|\ell|}, \tag{S5}$$

where γ_S is determined by the experimental conditions; and a Lorentz distribution

$$|\lambda_{\ell_L}|^2 \propto \frac{1}{\pi\gamma_L\left(1+\frac{\ell^2}{\gamma_L^2}\right)}, \tag{S6}$$

*isaacnape@gmail.com

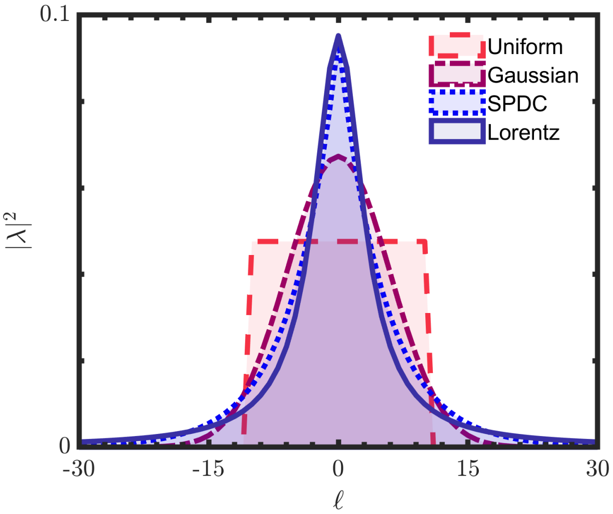

Supplementary Fig. 1: **Modal spectrum shapes.** Examples of various OAM (ℓ) distributions for a quantum source possessing OAM entanglement.

where γ_L is a scaling parameter.

For convenience, we relate the Schmidt number to the scaling parameters as, $\gamma_S \approx \sqrt{(K-1)/4}$ and $\gamma_G \approx 2.5066K$ for the SPDC and normal distributions, respectively. Later, we show how one can use the dimensional (K) together with purity to deduce the Schmidt rank (entanglement dimensionality) [3] for mixed states. Moreover these definitions are not restricted to the SPDC modes or OAM modes. We will in subsequent sections show how the technique can be extended to other degrees of freedom.

Supplementary Note 2 - High dimensional state projections

To introduce the projections required for our technique, we will describe them in the OAM basis and later generalise them to other degrees of freedom, i.e. the pixel basis. We take this approach since the projections were initially discovered in the OAM basis [6]. We can represent our analysers on the high dimensional Hilbert space using the OAM basis modes $|\ell\rangle \in \mathcal{H}_\infty$ as

$$|M,\alpha\rangle = \sum_{\ell=-\infty}^{\infty} c_{\ell,M}(\alpha)\,|\ell\rangle\,, \tag{S7}$$

where the complex coefficients, $c_{\ell,M}(\alpha)$, are computed from the overlap integral, $\int e^{-i\ell\phi}e^{i\Phi_M(\phi;\alpha)}\,d\phi$. Here $e^{i\Phi_M(\phi;\alpha)}$ is the azimuthally dependent mode characterizing the analyser orientated at an angle α. These modes project onto fractional OAM states [6]. Note that a complete decomposition would require an expansion onto a complete basis that includes the radial component. For brevity, we restrict ourselves to the azimuthal degree of freedom, consistent with [6].

By computing the overlap integral, one arrives at complex amplitudes

$$c_{\ell,M}(\alpha) = -\frac{ie^{-i\ell\alpha}\sin(\mu\pi)}{\pi(M-\ell)}, \tag{S8}$$

with μ representing the fractional part of the total charge M. The detection probability for each OAM mode with charge ℓ is therefore

$$P_\ell = |c_{\ell,M}(\alpha)|^2 = \frac{\sin^2(\mu\pi)}{\pi^2(M-\ell)^2}, \tag{S9}$$

consistent with probability amplitudes computed in [6] for fractional OAM states.

We have shown that fractional OAM modes project onto the high dimensional state space of OAM modes with complex amplitudes given by Supplementary Equation (S8). Next, we tailor new amplitudes and phases by superimposing rotated fractional OAM modes

$$|M,\alpha\rangle_n = \mathcal{N}\sum_{k=0}^{n-1}|M,\beta_k\oplus\alpha\rangle\,, \tag{S10}$$

where $\mathcal{N}$ is a normalization constant. Each fractional mode in this superposition has the same charge, M, but is rotated by an angle $\beta_k\oplus\alpha = \mathrm{mod}\,\{\beta_k+\alpha, 2\pi\}$, with $\beta_k = \frac{2\pi}{n}k$. In the OAM basis, Supplementary Equation (S10) becomes

$$\begin{aligned}|M,\alpha\rangle_n =& \mathcal{N}\sum_{k=0}^{n-1}\left\{\sum_\ell c_{\ell,M}(\beta_k\oplus\alpha)\,|\ell\rangle\right\},\\ =&\mathcal{N}\sum_\ell c^n_{\ell,M}(\alpha)\,|\ell\rangle\;,\end{aligned} \tag{S11}$$

where the coefficients $c^n_{\ell,M}(\alpha)$ are computed from

$$c^n_{\ell,M}(\alpha) = \sum_{k=0}^{n-1} c_{\ell,M}(\beta_k\oplus\alpha). \tag{S12}$$

Using Supplementary Equation (S8) and the condition mod $\{\beta_k\oplus\alpha, 2\pi\}=0$, we obtain

$$c^n_{\ell,M}(\alpha) = c_{\ell,M}(\alpha)\sum_{k=0}^{n-1}e^{i\beta_k\ell}. \tag{S13}$$

Since the summation can be evaluated as a geometric series, after some simplification it results in

$$\sum_{k=0}^{n-1}e^{i\beta_k\ell} = e^{-i\pi\ell(n-1)/n}\csc\left(\frac{\pi\ell}{n}\right)\sin(\pi\ell).$$

Therefore the coefficients can be written as

$$c^n_{\ell,M}(\alpha) = e^{-i\pi\ell(n-1)/n}A^n_\ell\;c_{\ell,M}(\alpha)\,, \tag{S14}$$

where

$$\begin{aligned}A^n_\ell =& \csc\left(\frac{\pi\ell}{n}\right)\sin(\pi\ell)\,,\\ =&\begin{cases}0 & \mathrm{mod}\,\{\ell,n\}\neq 0\\ 1 & \mathrm{mod}\,\{\ell,n\}=0\end{cases}.\end{aligned} \tag{S15}$$

Consequently, the overlap probabilities are $P_{\ell,n} = |\mathcal{N}\;A^n_\ell c_{\ell,M}(\alpha)|^2$. Importantly, the probabilities are independent of α. Accordingly, the new spectrum has the amplitudes $|c_{\ell,M}|$, but following the selection rule A^n_ℓ. Indeed, this new spectrum can be tuned by carefully selecting n, therefore enabling control of the OAM subspaces.

Supplementary Note 3 - Decomposition of Entangled Photons

Our fractional OAM analysers can be decomposed into the OAM basis using entangled photons through digital spiral imaging. In this scheme, one photon from an entangled pair interacts with the analyser while its twin is decomposed in the OAM basis. The entangled photon pair has a biphoton state

$$|\Psi\rangle = \sum_{\ell=-L}^{L}\lambda_\ell\,|\ell\rangle\,|-\ell\rangle\,. \tag{S16}$$

The probability amplitude for detecting the mth OAM mode, given a M charged fractional mode of n superpositions, is

$$\begin{aligned}\tilde{c}^n_m(\alpha) =& \mathcal{M}\,\langle m|\,\langle M,\alpha|_n|\Psi\rangle\,,\\ =&\mathcal{M}\sum_{\ell=-L}^{L}\lambda_\ell\,\langle m|\ell\rangle\,\langle M,\alpha|_n|-\ell\rangle\,.\end{aligned} \tag{S17}$$

where $\mathcal{M}$ is a normalisation constant such that $\sum_m|\tilde{c}^n_m(\alpha)|^2 = 1$. Due to the orthonormality of the OAM basis, the overlap $\langle m|\ell\rangle$ is simply the Kronecker delta function $\delta_{m,\ell}$, which evaluates as 0 if $\ell\neq m$ or 1 if $\ell=m$. Since from Supplementary Equation (S14) we know the expansion coefficients for the analyser in terms of the OAM basis, $\langle M,\alpha|_n|\ell\rangle$ evaluate as

$$\begin{aligned}\tilde{c}^n_m(\alpha) =& \mathcal{M}\sum_{\ell=-L}^{L}\delta_{m,\ell}\lambda_\ell\;\left[\mathcal{N}c^n_{-\ell}(\alpha)\right]^*\\ =&\mathcal{M}\mathcal{N}\;\lambda_m\;\left[c^n_{-m}(\alpha)\right]^*.\end{aligned} \tag{S18}$$

These new weightings are simply the original coefficients of the analysers modulated by the spectrum of the entangled system. For a maximally entangled state, we obtain the expression $|\tilde{c}^n_m(\alpha)|^2 = |c^n_{-m}(\alpha)|^2$, being the original weightings of the analyser, as desired.

In Supplementary Fig. 2 we show the measured weightings for our SPDC system which has a normal distribution of OAM modes with $\Delta\ell = 11$ centered at $\ell = 0$. We show results for $|M,\alpha\rangle_n =$

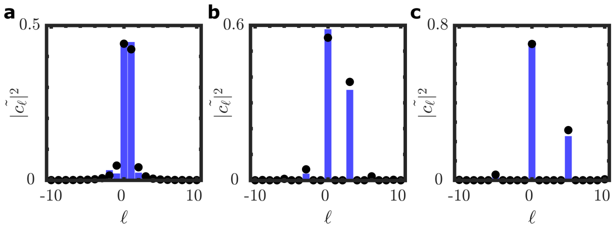

Supplementary Fig. 2: **Modal decomposition of the state projectors.** Measured (bars) and theoretical spectrum (points) for fractional OAM analysers (a) $|M,\alpha\rangle_n = |0.5,0\rangle_1$, (b) $|M,\alpha\rangle_n = |1.5,0\rangle_3$, and (c) $|M,\alpha\rangle_n = |2.5,0\rangle_5$ resulting from digital spiral imaging with entangled photons. Here the weightings are modulated by the OAM spectrum of the entanglement source according to Supplementary Equation (S18).

$|0.5,0\rangle_1, |1.5,0\rangle_3, |2.5,0\rangle_5$ for analysers $n = 1,3$ and 5 in Supplementary Fig. 2(a), (b) and (c), respectively. It can be seen that the theory (points) and experiment (bars) are in good agreement. To obtain these results, two photons where generated from an SPDC source and modulated with SLMs (see experimental setup in the Methods section in the main text). One SLM was encoded with a fractional OAM mode projecting onto the state, $|M,0\rangle_n$, while the second SLM was encoded with OAM basis modes, $|\ell\rangle$.

Supplementary Note 4 - Detection Probability

Given a bipartite system of the form of Supplementary Equation (S16), we want to know what the detection probability is, due to the relative rotations of our fractional OAM analysers acting on the entangled photons. Suppose the first analyser projects onto the state $|M,\theta_1\rangle_n$, and the second analyser projects on the state $|-M,\theta_2\rangle_n$. A joint measurement on a two photon system using the two analysers is characterized by the product state $|M,\theta_1\rangle_n |-M,\theta_2\rangle_n$. The probability amplitude resulting from such a measurement is

$$\begin{aligned} C_n(\theta_1,\theta_2) &= \langle\theta_2,-M|_n \langle\theta_1,M|_n|\Psi\rangle \\ &= \sum_{\ell=-\infty}^{\infty} \lambda_\ell \ \langle\theta_1,M|_n|\ell\rangle \ \langle\theta_2,-M|_n|-\ell\rangle \,. \end{aligned} \tag{S19}$$

Therefore we only need to know how to decompose each of the analysers in the OAM basis to obtain the detection probability for the joint measurements. Using Supplementary Equation (S14), it follows that

$$C_n(\theta_1,\theta_2) \propto \sum_{\ell=-\infty}^{\infty} \underbrace{\lambda_\ell}_{\text{SPDC}} \ [\ \underbrace{c^n_{\ell,M}(\theta_1)}_{\text{analyser}} \ \underbrace{c^n_{-\ell,-M}(\theta_2)}_{\text{analyser}}\ ]^*. \tag{S20}$$

We use this approach to numerically calculate the detection probabilities $|C_n(\theta_1,\theta_2)|^2$ by simply calculating the probability amplitudes for each analyser in the OAM basis with a desired rotation $\theta_{1,2}$ and multiplying them with the coefficients λ_ℓ that determine the quantum system being probed.

An alternative approach, can be to compute the overlap integral by considering the modal overlaps in the azimuthal degree of freedom, ϕ, following

$$\langle\theta,M|_n|\ell\rangle = \frac{1}{2\pi}\int \exp(-i\Phi_M(\phi;\theta)) \times \exp(i\ell\phi)\ d\phi,$$

with $\Phi_M(\phi;\theta)/\sqrt{2\pi}$ being the transmission function of the fractional OAM analyser projecting onto the state $|M,\theta\rangle_n$. We can rewrite the probability amplitude $C_n(\theta_1,\theta_2)$ as an overlap integral given by

$$\begin{aligned} C_n(\theta_1,\theta_2) = \frac{1}{4\pi}\sum_{\ell=-\infty}^{\infty}\Bigg(\lambda_\ell \iint e^{-i\Phi_M(\phi_1;\theta_1)} e^{i\ell\phi_1} \\ \times e^{-i\Phi_{-M}(\phi_2;\theta_2)} e^{-i\ell\phi_2}\ d\phi_1 d\phi_2\Bigg), \end{aligned} \tag{S21}$$

where $\Phi_{\pm M}(\phi_{1,2},\theta_{1,2})$ are the phases of the fractional OAM analysers. Since $e^{-i\Phi_M(\phi_1;\theta_1)}$ has no ℓ dependence, we can introduce the summation into the second integral resulting in

$$\begin{aligned} C_n(\theta_1,\theta_2) = \frac{1}{2\pi}\int e^{-i\Phi_M(\phi_1;\theta_1)} \Bigg(\int e^{-i\Phi_M(\phi_2;\theta_2)} \\ \times\frac{1}{2\pi}\sum_{\ell=-\infty}^{\infty}\lambda_\ell e^{i\ell(\phi_1-\phi_2)} d\phi_2\Bigg) d\phi_1. \end{aligned} \tag{S22}$$

It is convenient to define the periodic function

$$\Lambda(\phi_1-\phi_2) = \frac{1}{2\pi}\sum_{\ell=-\infty}^{\infty}\lambda_\ell e^{i\ell(\phi_1-\phi_2)},$$

with angular harmonics $e^{i\ell(\phi_1-\phi_2)}$ determined by the coefficients λ_ℓ, and use it to rewrite $C_n(\theta_1,\theta_2)$ as

$$\begin{aligned} C_n(\theta_1,\theta_2) = \frac{1}{2\pi}\int e^{-i\Phi_M(\phi_1;\theta_1)} \\ \left(\int e^{-i\Phi_M(\phi_2;\theta_2)}\Lambda(\phi_1-\phi_2) d\phi_2\right) d\phi_1. \end{aligned} \tag{S23}$$

Notice that the second integral is a convolution between $\Lambda(\phi_1-\phi_2)$ and the second analyser. As a example, we consider a maximally entangled state ($\lambda_\ell :=$ constant). In this case, $\Lambda(\phi_1-\phi_2) = \delta(\phi_1-\phi_2)$ and therefore

$$C_n(\theta_1,\theta_2) = \frac{1}{2\pi}\int e^{-i\Phi_M(\phi;\theta_1)}\ e^{-i\Phi_{-M}(\phi;\theta_2)} d\phi.$$

The integral now only depends in the transmission functions of the analysers with an analytical solution found in [7].

We now calculate the probability $P_n(\theta_1,\theta_2) = |C_n(\theta_1,\theta_2)|^2$ as a function of relative orientation $\theta = (\theta_1-\theta_2)$ between the two analysers and the dimensions, K, of an entangled system with some given OAM

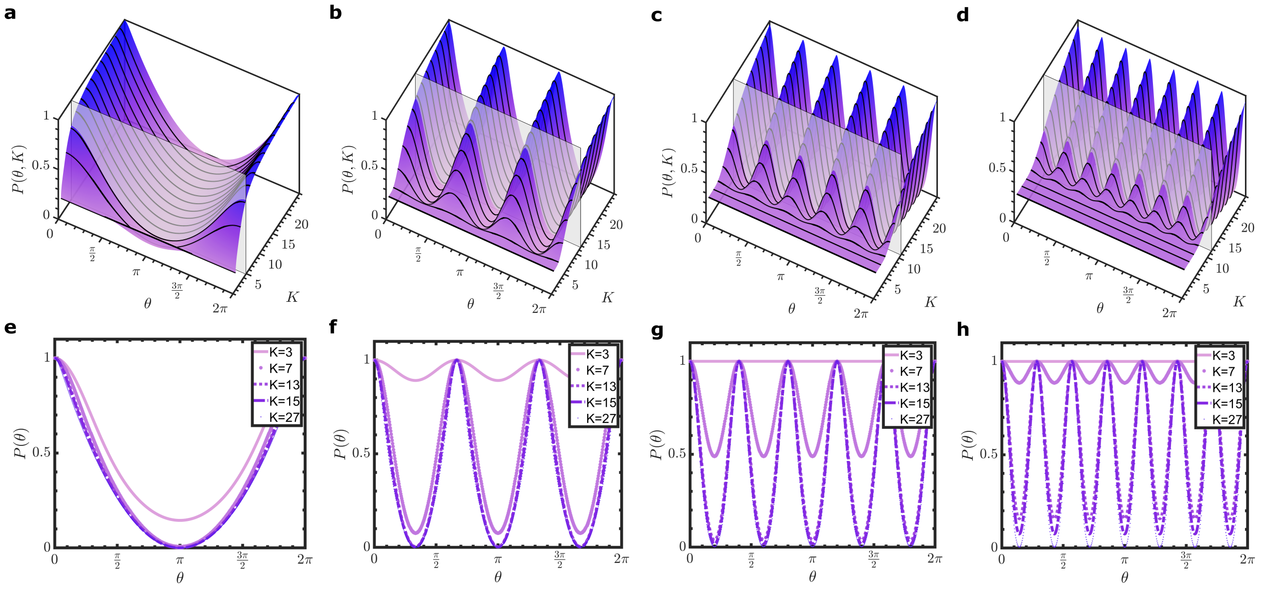

Supplementary Fig. 3: **Detection probability vs relative orientation and dimensionality.** Simulated normalised probability curves for (a) $n = 1$, (b) $n = 3$, (c) $n = 5$ and (d) $n = 7$, with an analyser corresponding to $M = \frac{n}{2}$, as a function of the relative orientation θ between the two analysers and the dimensions, K, of the entanglement state. The bottom row of panels are probability curves for specific K values for each analyser. The normalisation to unity was performed to illustrate the impact of the dimensions on the visibility. Here, the OAM spectrum shape was assumed to follow a normal (Gaussian) distribution.

spectrum $|\lambda_\ell|^2$. The latter is embedded in the function $\Lambda(\phi_1 - \phi_2)$. Supplementary Figures 3(a)-(d) show examples of the probability surfaces assuming a normal (Gaussian) spectrum $|\lambda_\ell|^2$ for superposition states $n = 1, 3, 5$ and 7. In the second row of Supplementary Fig. 3, we show examples of the probability curves normalised to unity for several values of dimensionality K. Here, it can be seen that the frequency of the probabilities as a function of θ increases with n, owing to the n-fold symmetry in the phase profiles of the analysers.

Crucially, the exact shape and visibility of the curves depends on both the dimensions (K) of the state being probed and the number of superpositions (n). For all n's, the visibility for a specific K shows a decreasing trend as the number of superpositions n are increased. Therefore the analysers are sensitive to the dimensions of the system.

We also found that the shape of the spectrum affects the measured probabilities, as illustrated in Supplementary Fig. 4(a)-(c) for the normal (Gaussian), SPDC and square (maximally entangled) distributions, respectively.

Now that we have shown how the detected probabilities depended on the dimensions and superposition states measured, in the following section we study the relation between the visibility and dimensions quantitatively.

Supplementary Note 5 - Visibility for Different Spectra

The visibilities are calculated from detection probabilities resulting from the projections of an entangled state with an initial OAM distribution $|\lambda_\ell|^2$ onto the states $|M, 0\rangle_n |-M, \theta\rangle_n$, where $\theta \in [0, 2\pi]$ is their relative rotation.

For example, for a square (uniform) ($K \to \infty$) distribution and n superpositions of fractional modes the probability is given by [7]

$$\begin{aligned} P(\theta_1, \theta_2) =& |C(\theta_1, \theta_2)|^2 \\ =& a \sin^2\left(\frac{M\pi}{n}\right) + \cos^2\left(\frac{M\pi}{n}\right), \end{aligned} \quad \text{(S24)}$$

with $a = (\pi(2t-1) - n\theta)^2/\pi^2$ for $\frac{2\pi}{n}(t-1) \leq \theta \leq \frac{2\pi}{n}(t), \quad t = 1, ..., n$, where t indexes each $2\pi/n$ period over the range of $0 \leq \theta < 2\pi$ and $\theta = \theta_1 - \theta_2$. This oscillating function results in fringes with a visibility function given by

$$V_n(M) = \frac{1 - \cos^2\left(\frac{M\pi}{n}\right)}{1 + \cos^2\left(\frac{M\pi}{n}\right)}.$$

For $n = 1$, parabolic fringes with perfect visibility occur when $M = \ell + 0.5$ for all OAM integer charges ℓ. In contrast, when $n > 1$ high visibility fringes occur for only specific choices of n and M. That is, parabolic fringes with high visibility ($V = 1$) are expected when n is odd and mod $\{M - \frac{n}{2}, n\} = 0$.

Contour plots of the visibilities with changing dimensions (K) and fractional OAM superpositions (n) for various OAM spectral shapes (Normal, SPDC, Uniform) are shown in Supplementary Fig. 4 (a)-(c) for pure states. As shown, the assumed spectrum can affect the visibility that is measured for various superpositions (n). The visibilities for each analyser (n) and spectrum shape are monotonic with increasing K. In

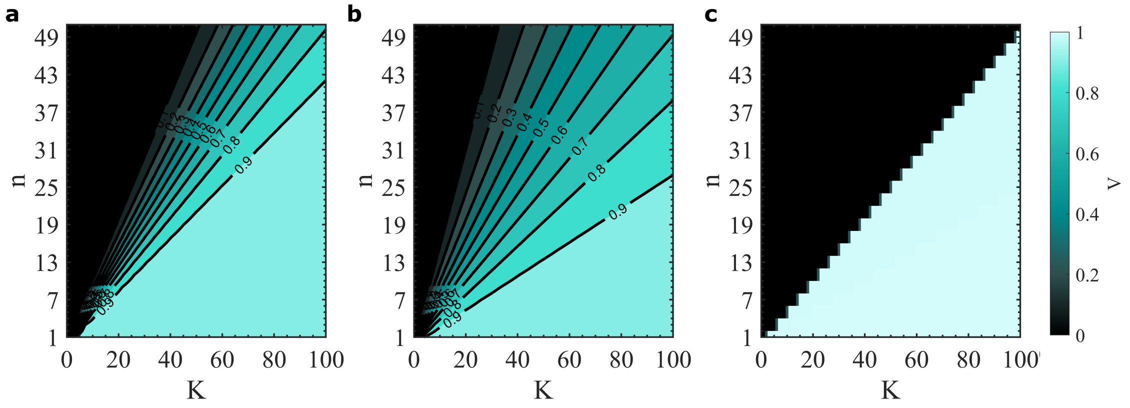

Supplementary Fig. 4: **Visibility of entangled pure states with differing spectral shapes.** Contour plots of visibility vs dimensionality (K) and n (indexing the projection state $|M,\theta\rangle_n$) for the (a) Normal, (b) SPDC theory and (c) maximally uniform distribution (or maximally entangled pure state). Here we demonstrate the sensitivity of the analysers to the dimensions of a OAM entanglement. The visibilities from the maximally entangled state demonstrates the minimum number of modes required to have a visibility $V = 1$.

particular, for the uniform spectrum (maximal entanglement in K dimensions) the visibility is 1 above some $K = d_n$ and zero below this. We further exploit this property to determine the dimensionality of an entanglement system.

Supplementary Note 6 - Visibility of Mixed States

The visibilities that can be measured with our analysers are not only dependent on the effective dimensions of the system but also the purity. In particular, we consider the isotropic state,

$$\rho_p = p\,|\Psi_d\rangle\langle\Psi_d| + \frac{1-p}{d^2}\mathbb{I}_{d^2}, \tag{S25}$$

which can decomposed into the high-dimensional entangled state, $|\Psi_d\rangle$, and the separable and mixed state, $1/d^2\mathbb{I}_{d^2} = 1/d^2\sum_{\ell,\ell'=-L}^{\ell,\ell'=L}|\ell\rangle|\ell'\rangle\langle\ell'|\langle\ell|$, where $\mathbb{I}_{d^2}$ is the identity operator. Such states model quantum systems that have noise contributions from the environment. Here p can be associated with the purity of the state ranging from a maximally mixed ($p = 0$) to a pure state ($p = 1$). Interestingly, the isotropic state is separable for $p \leq 1/(d+1)$ and entangled otherwise. Importantly, the generalised Bell inequality can also be violated when $p > 2/S_d$ where S_d is the Bell parameter [8]. We show that both p and d can be measured using our analysers. For convenience, we assume $d \approx K$, where K is the effective dimensionality of the pure state. We will demonstrate that we can measure both p and K using our analysers.

Firstly, we calculate the detection probabilities from the overlap, $P_n(\theta; K, p) = \text{Trace}(\hat{M}\rho_p)$ where $\hat{M}$ projects onto the states $|M,0\rangle_n|-M,\theta\rangle_n$. As a result, the detection probability can be written as

$$P_n(\theta; p, K) = pP_n(\theta; K) + \frac{1-p}{K^2}I_n(0; K), \tag{S26}$$

where $P_n(\theta, K) = \left|\sum_{\ell=-L}^{L}\lambda_\ell c_\ell^n(0)c_{-\ell}^n(\theta)\right|^2$ and $I_n(0; K) = \left|\sum_{\ell=-L}^{L}|c_\ell^n(0)|^2\right|^2$ is the overlap of the analysers with the maximally mixed state. Since the functions are periodic and obtain maximum and minimum values for $\theta = 0$ and π/n, respectively, we obtain the expression

$$\begin{aligned}\Delta P_n(p, K) &= P_n(0; p, K) - P_n(\pi/n; p, K)\\ &= p\Delta P_n(K),\end{aligned} \tag{S27}$$

where $\Delta P_n(K) = P_n(0, p = 1, K) - P_n(\pi/n, p = 1, K)$. The visibilities can be calculated from

$$V_n(p, K) = \frac{\Delta P_n(p, K)}{P_n(\pi/n; p, K) + P_n(0; p, K)}, \tag{S28}$$

We show the dependence of the visibilities on the dimensions (K) and purity (p) in Supplementary Fig. 5 (a-c) for the Normal, SPDC and uniform distribution, respectively. Each panel shows the visibilties from various analysers depending on the number of superpositions (n). As shown the visibilities increase monotonically with increasing dimensions (K) as well as purity p for each analyser. However, as n increases the visibilities decrease for all p and K. Since the visibilities are monotonic in both p and K as well as n, we can exploit this property to map the dimensions of a quantum system. We favour this approach since the visibilities can be easily measured and require few measurements (peak and trough).

Supplementary Note 7 - Visibility of Separable States

Consider a system that is completely separable,

$$\rho = 1/D\sum_{\ell_1\ell_2}|\ell_1\rangle|\ell_2\rangle\langle\ell_2|\langle\ell_1|, \tag{S29}$$

where the product states, $|\ell_1\rangle|\ell_2\rangle$, are orthogonal. We will show that such a state yields a visibility of

zero. Firstly, the overlap probability of Supplementary Equation (S29) with our analyser projecting onto the state $|M,0\rangle_n |-M,\theta\rangle_n$, is

$$P(\theta) = \langle\theta,-M|_n \langle 0,M|_n \rho |M,0\rangle_n |-M,\theta\rangle_n \quad \text{(S30)}$$

$$\propto \sum_{\ell_1,\ell_2} |c^n_{\ell_1,M}(0)|^2 |c^n_{\ell_2,M}(\theta)|^2, \quad \text{(S31)}$$

which follows from Supplementary Equation (S11). Since, from Supplementary Equation S8, $|c^n_{\ell_2,M}(\theta)|^2 = |c^n_{\ell_2,M}(0)|^2$, Supplementary Equation S31 simplifies to

$$P(\theta) \propto (\sum_{\ell_1} |c^n_{\ell_1,M}(0)|^2)^2, \quad \text{(S32)}$$

We see that $P(\theta) = P(0)$ for all θ, meaning that $|P(0) - P(\pi/n)| = 0$. This implies that the visibility of separable mixed states is always 0.

Supplementary Note 8 - Verification of the Technique

Using our procedure we measured the dimensions and purity of SPDC photons with varying noise levels (low and high). The results are summarised in Supplementary Table 1. In the second and third column, we know what the input spectrum shape is (SPDC) and can therefore accurately optimise for the dimensions (K) and purity (p) of the state (see Results section). Further, if we guess the spectrum based on its shape (symmetry) we also obtain values that are similar to the expected results, with a relative error of up to $\approx 13\%$. This was done using the normal distribution as the function modelling the mode spectrum. Next, we verify our result using the values extracted from the spiral bandwidth.

To calculate the expected dimensions, $\hat{K}$, we used the coincidences from the spiral spectrum in the OAM basis, i.e C_{ℓ_A,m_B}, where ℓ_A denotes the mode index of photon A and m_B for photon B. Since we want the Schmidt number of the pure part of the state, we subtracted the accidentals and then used Supplementary Equation (S2), yielding results with a low relative error of 3%, validating our results. Subsequently, we estimated the purity $\hat{p}$ (see Results section). Note that no accidentals subtraction was performed in this case. Accordingly, to estimate the purity, we measured the quantum contrast using

$$Q = \bar{C}/\bar{C}', \quad \text{(S33)}$$

taken from the ratio between the average coincidences in the anti-diagonal entries, $\bar{C} = \sum_\ell C_{\ell,-\ell}$ and the average noise contribution from coincidences excluding the anti-diagonal entries, i.e $\bar{C}' = \frac{1}{d^2-d}\left(C_T - d\,\bar{C}\right)$. Here C_T corresponds to the total coincidences $C_T = \sum_{\ell,m} C_{\ell,m}$. Indeed, using the quantum contrast we obtained a purity that is comparable to that obtained from our method showing a relative error of only up to 2%.

Supplementary Note 9 - Simulations in the Pixel Basis

We demonstrate our technique using the pixel basis. Firstly, we define an entangled state using the pixel position basis modes, $|j\rangle$ for $\{j = 1, 2...\}$. Since we are modelling the isotropic state in Supplementary Equation (S25), we define the pure part of the state as

$$|\Psi_d\rangle = \sum_{j=0}^{d-1} \lambda_j |j\rangle |j\rangle \quad \text{(S34)}$$

on a d-dimensional space. Our analysers are now defined as

$$|M,\alpha\rangle_n = \mathcal{N} \sum_{j=0}^{d-1} c^n_{w_j,M}(\alpha) |j\rangle, \quad \text{(S35)}$$

composed of coherent superpositions of basis states $|j\rangle \in \{|j\rangle, j = 0, 1..d-1\}$ with tune-able phases and amplitudes

$$c^n_{w_j,M}(\alpha) = e^{-i\pi w_j (n-1)/n} A^n_{w_j} c_{w_j,M}(\alpha), \quad \text{(S36)}$$

and where $w_j = j - (d-1)/2$ and the factors

$$c_{w_j,M}(\alpha) = -\frac{ie^{-iw_j\alpha}}{\pi(M - w_j)}. \quad \text{(S37)}$$

and

$$A^n_{w_j} = \begin{cases} 1, & \mathrm{mod}\,\{w_j, n\} = 0 \\ 0, & \text{otherwise} \end{cases}. \quad \text{(S38)}$$

The projections follow the same procedure as in the OAM basis, i.e., projections onto the states $|M,0\rangle_n |-M,\theta\rangle_n$ at angles $\theta = 0$ and π/n. Subsequently, the visibilities can be measured from these two projections for each nth analyser.

Examples of simulations for χ^2 over the parameter space (K, p) are shown in Supplementary Fig. 6 with the maximally entangled state.

Supplementary Note 10 - Quantum State Fidelity and Schmidt Rank

Using our technique it is possible to determine how well the state, parametrised by K and p, generally approximates a maximally entangled state $|\Phi\rangle = \sum_i^d \gamma_i |i\rangle |i\rangle$ and how it generally performs with respect to well known entanglement witnesses.

Firstly, we note that our definition of the dimensionality (K), generally estimate the effective number of Schmidt modes required to describe a pure state and therefore reflects how large the Hilbert space is. From Supplementary Fig. 7 we see that K maintains a high value for very low values of p meaning that our measurement technique can sift out the dimensionality of the pure part of the state under extremely noisy conditions. As such, in the isotropic state, it gives us an

Noise level	p^{SPDC}	K^{SPDC}	p^{normal}	K^{normal}	Q	$\hat{K}$	$\hat{p}$
low	0.45 ± 0.03	22.84 ± 0.62	0.42 ± 0.02	20.00 ± 0.32	19.19 ± 0.22	22 ± 1	0.44 ± 0.01
high	0.13 ± 0.01	17.73 ± 0.71	0.13 ± 0.01	17.18 ± 0.34	3.76 ± 0.57	18 ± 1	0.13 ± 0.02

Supplementary Table 1: **Dimensionality and purity measurements in the OAM basis.** Measured purity (p) and dimensionality (K), under low and high noise levels, compared to estimates from other methods. Here Q is the average quantum contrast.

indication of the number of modes that posses strong correlations, given a purity (probability) p.

To relate our measured dimensionality (K) with a common entanglement witness, we consider the operational definition of Schmidt rank, k [3], in comparison to our method. Firstly, the operational general definition of Schmidt rank [2], is the k for which

$$F(\rho, \Phi) \leq B_k(\Phi) \tag{S39}$$

where $F(\rho, \Phi) = \mathtt{Tr}(\rho, |\Phi\rangle\langle\Phi|)$ is the fidelity while $B(\Phi) = \sum_i^k |\gamma_i|^2$. For a maximally entangled state, $B(\Phi) = k/d$. Therefore one finds the lower bound of the Schmidt rank $k-1$ when $F(\rho, \Phi) > B_k(\Phi)$ [3, 2]. For the isotropic state, the Schmidt rank is determined by $d_{ent} := dF(\rho, \Phi)$ [3]. Since the relation between the fidelity (F) and purity (p) is known, i.e.,

$$F_p = \frac{p(d^2-1)+1}{d^2} \tag{S40}$$

we can estimate the fidelity of the state using our measured $d = K$ and as well as p. This means that for a state with an effective dimensionality of $d = K$, the Schmidt rank is given by $d_{ent} \approx KF_p$ [2]. We show both the entanglement dimensionality from [2] and our approximation $K * F_p$ in Supplementary Fig. 7, which are in good agreement.

Supplementary Note 11 - Measurements in the Pixel Basis

In pixel basis we projected the photons onto the states $|M, 0\rangle_n |-M, \theta\rangle_n$ for $\theta = 0, \pi/n$ and n = 1, 3, 5...$(d-1)/2+1$. From the measured probabilities we calculated the visibilities and applied the optimazation procedure to determine p and K. Here the model spectrum of the source was assumed to be normally distributed over the pixels.

We demostrate the technique over square pixel states from 3×3, 5×5, 7×7, 9×9 and 11×11 grids constituting test dimensions of d = 9, 25, 49, 81 and 121, respectively. For the first three cases we varied the laser power in order to change the purity of the state for average photon numbers of μ = 0.002, 0.003 and 0.01 with an integration time of 10 seconds per measurement. For the 11×11 states, we had an average photon number of $\mu = 0.001$ while we used an integration time of 20 second per measurement. In this case the 11 projections produced p = 0.01 ± 0.004 and K = 119 ± 1 witout accidental subtraction. After accidental subtraction we obtained, p = 0.04 ± 0.002 and K = 121 ± 2. The purity in these cases was above the separability bound of $p = 0.0082$.

The results are shown in Supplementary Fig. 8 for the first three cases of grid states. Here, the dimensions (K) of the pure part of the state increases as the average photon number in the system decreases due to the reduction in the number of multi-photon events from the SPDC source. The purity is also seen to improve showing that the measurement technique is sensitive to multi-photon events and noise in the system. The entanglement dimensions, do not seem to improve due the low purity in the system. This can be explained by the fact that as the dimensions of the system increase, a much higher purity is needed to ensure d-dimensional entanglement.

Supplementary Note 12 - Comparison to State-of-the-art

We compare the number of measurement needed in our approach for determining the dimensionality and purity of a quantum state in the table 2. As compared to QST and the recent two MuB approach, the number of measurements are far fewer; in our approach the number measurements scale linearly rather than quadratically for the other two approaches. This can significantly reduce the measurement time of quantum state characterisation.

		QST	2 MUB
No. of analysers	$\frac{d+1}{4} \sim \frac{d}{4}$	d^2 - 1	$2d$
Local proj.	$\sim \frac{d}{2}$	$(d^2-1)d^2$	$2d^2$

Supplementary Table 2: **Measurement scaling with dimensions.** Comparison of our technique to the traditional QST [9] and two MuB approach [2].

References

[1] C. Law and J. Eberly, "Analysis and interpretation of high transverse entanglement in optical parametric down conversion," *Phys. Rev. Lett.*, vol. 92, no. 12, p. 127903, 2004.

[2] J. Bavaresco, N. H. Valencia, C. Klöckl, M. Pivoluska, P. Erker, N. Friis, M. Malik, and M. Huber, "Measurements in two bases are sufficient for certifying high-dimensional entanglement," *Nat. Phys.*, pp. 1745–2481, 2018.

[3] B. M. Terhal and P. Horodecki, “Schmidt number for density matrices,” *Phys. Rev. A.*, vol. 61, no. 4, p. 040301, 2000.

[4] F. M. Miatto, A. M. Yao, and S. M. Barnett, “Full characterization of the quantum spiral bandwidth of entangled biphotons,” *Phys. Rev. A.*, vol. 83, no. 3, p. 033816, 2011.

[5] J. Torres, A. Alexandrescu, and L. Torner, “Quantum spiral bandwidth of entangled two-photon states,” *Phys. Rev. A.*, vol. 68, no. 5, p. 050301, 2003.

[6] J. B. Götte, S. Franke-Arnold, R. Zambrini, and S. M. Barnett, “Quantum formulation of fractional orbital angular momentum,” *J. Mod. Opt.*, vol. 54, no. 12, pp. 1723–1738, 2007.

[7] H.-C. Huang, “Various angle periods of parabolic coincidence fringes in violation of the bell inequality with high-dimensional two-photon entanglement,” *Phys. Rev. A.*, vol. 98, p. 053856, Nov. 2018.

[8] D. Collins, N. Gisin, N. Linden, S. Massar, and S. Popescu, “Bell inequalities for arbitrarily high-dimensional systems,” *Phys. Rev. Lett.*, vol. 88, p. 040404, Jan. 2002.

[9] M. Agnew, J. Leach, M. McLaren, F. S. Roux, and R. W. Boyd, “Tomography of the quantum state of photons entangled in high dimensions,” *Phys. Rev. A.*, vol. 84, p. 062101, 2011.

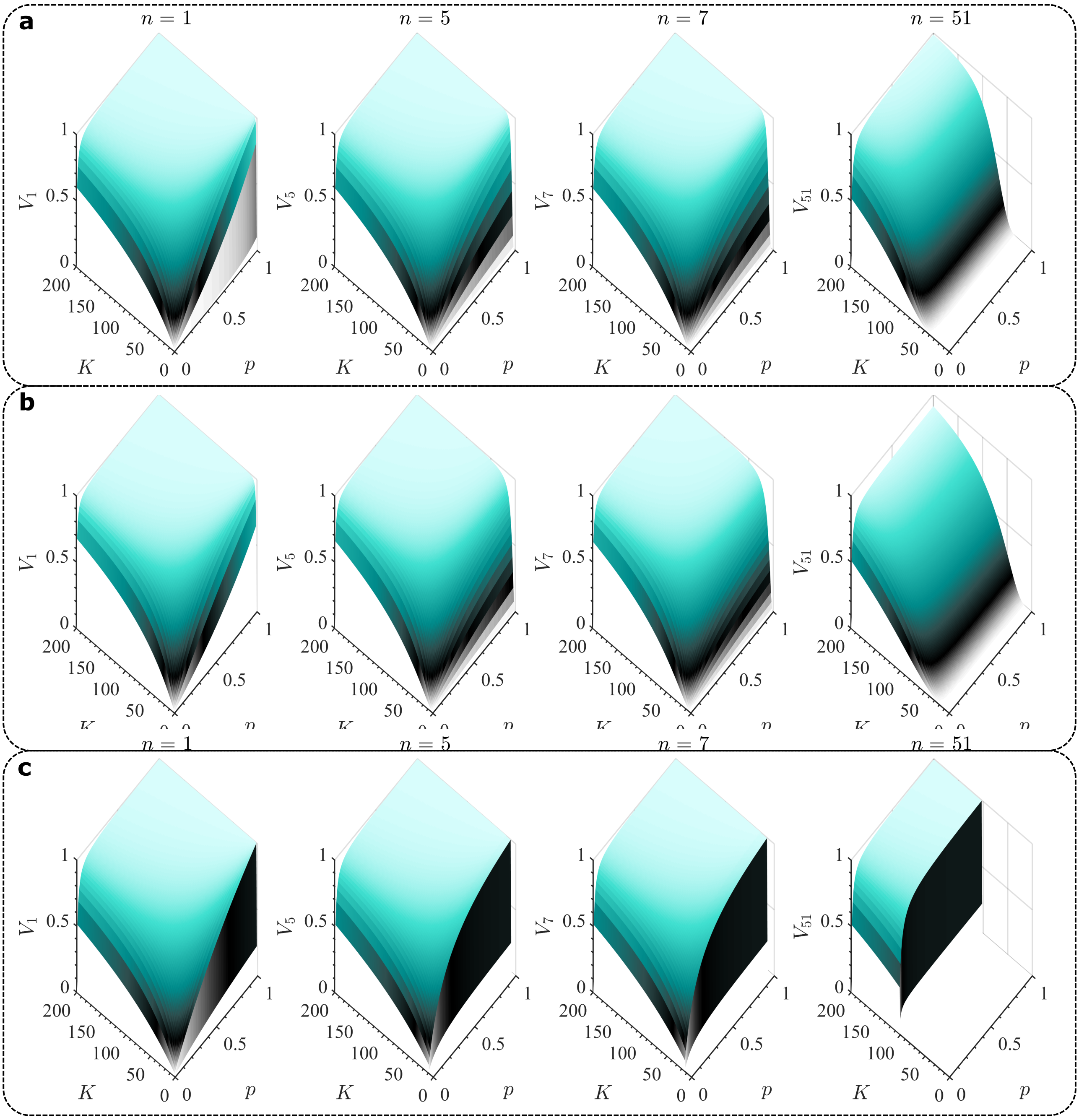

Supplementary Fig. 5: **Impact of purity and dimensionality on visibility.** Visibility as a function of purity (p) and dimensions (K) for the (a) Normal, (b) SPDC theory and (c) the uniform (maximally entangled state) obtained for fractional OAM projections corresponding to $n = 1, 5, 7, 51$.

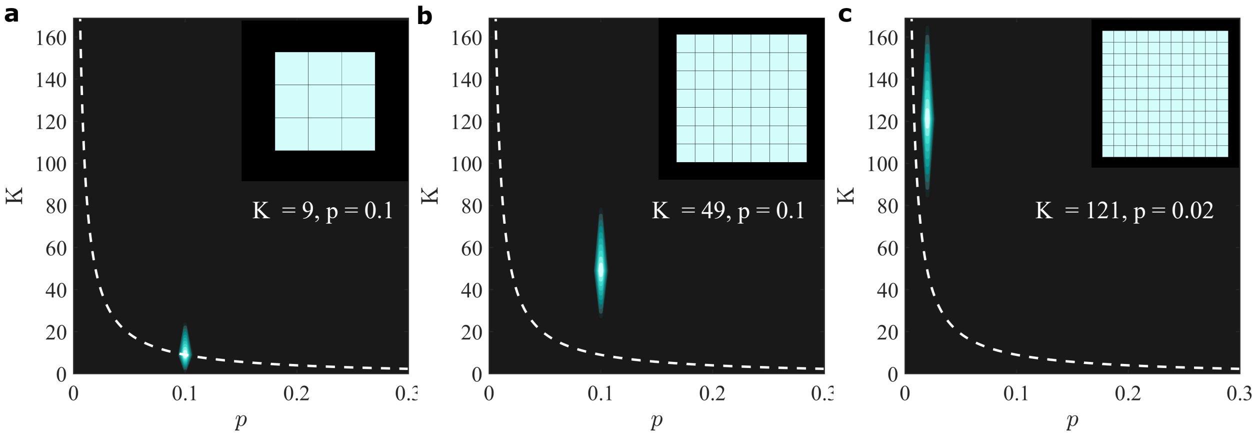

Supplementary Fig. 6: **Simulations for the pixel basis.** Simulated χ vs K and p for input states with dimensions $d = 9, 49$ and 121. The white dashed line defines the boundary between the separable and entangled states.

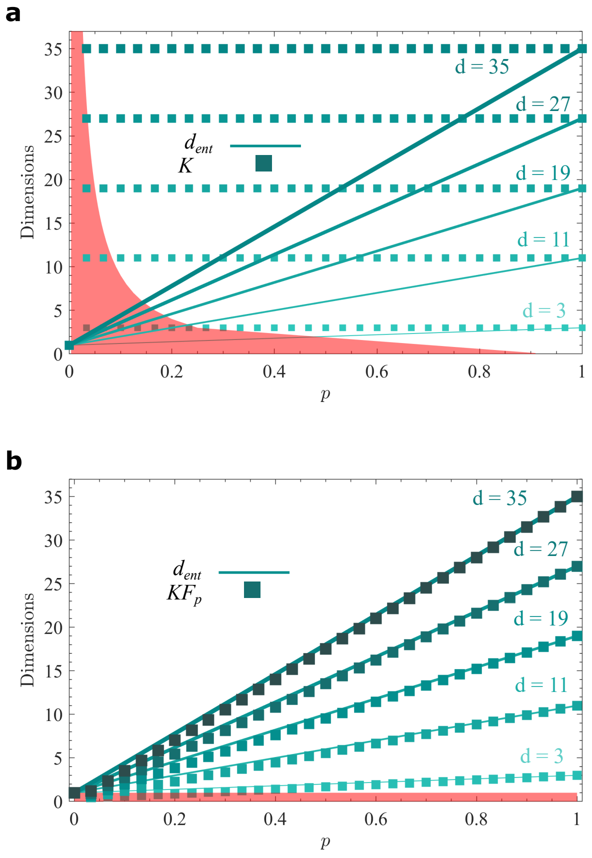

Supplementary Fig. 7: **Dimensionality witness.** On the top panel we show simulations of calculated entanglement dimensions using the our approach, K, and the Schmidt rank, $d_{ent} = d \times F$, as a function of purity, p. In the bottom panel we show d_{ent} and our estimation as computed from $K \times F_p$, which are in good agreement.

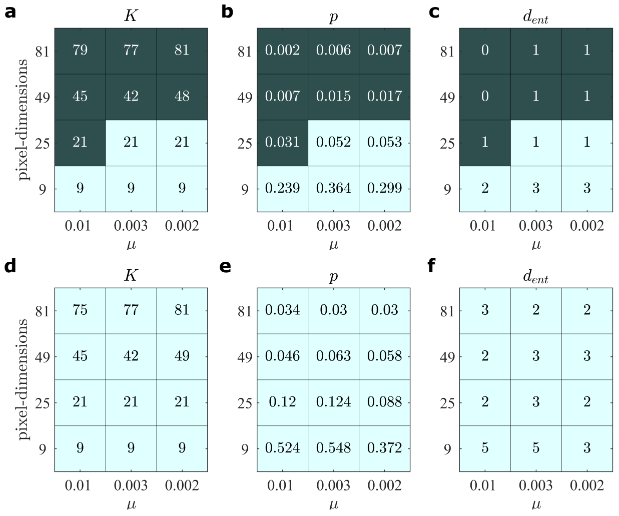

Supplementary Fig. 8: **Dimensionality and purity measurements in the pixels basis.** Experimental data for measurements of dimensionality (K) and purity (p) in the pixel basis for various average photon numbers (μ). (a) - (c) are projections with different pixels dimensions (grids), without noise subtraction, while (d) - (f) have accidental noise subtraction. The light squares mark results for purities above the separability bound, $1/(K+1)$.
